# The efficacy of using metformin and/or quercetin for amelioration of gamma-irradiation induced tongue toxicity in diabetic rats

**DOI:** 10.1186/s12903-024-03871-0

**Published:** 2024-01-18

**Authors:** Salwa Farid Ahmed, Mostafa A. Bakr, Amr H. Rasmy

**Affiliations:** Health Radiation Research Dept, National Center for Radiation Research and Technology, Egyptian Atomic Energy Authority, Cairo, Egypt

**Keywords:** Gamma-radiation, Diabetes, Histomorphometry, CD68, Metformin, Quercetin

## Abstract

**Background:**

Diabetes is a common disease that cancer patients may suffer from and may aggravate side effects of radiotherapy. This study aimed to detect whether metformin and/or quercetin will improve gamma-irradiation induced tongue toxicity in diabetic rats.

**Methods:**

35 male albino rats were divided into five groups; NOR no streptozotocin, no radiation and no treatment was given, DR rats were subjected to streptozotocin then gamma-irradiation, DRM rats were subjected to streptozotocin then gamma-irradiation then metformin, DRQ rats were subjected to streptozotocin then gamma-irradiation then quercetin, DRMQ rats were subjected to streptozotocin then gamma-irradiation then metformin and quercetin. Rats were euthanized 24 h after last treatment dose. Mean blood glucose level was recorded. Tongue specimens were stained with H&E and CD68. Histomorphometric analysis of length, diameter and taste buds of lingual papillae and epithelial, keratin and lamina propria thickness and CD68 positive cells were calculated.

**Results:**

Blood glucose level of DRMQ was significantly lower than DR, DRM and DRQ, whereas higher than NOR. Metformin or quercetin partially restored tongue structure, papillae length and diameter and tongue layers thickness. The ameliorative effect was superior when metformin and quercetin were used together. Diabetes and irradiation significantly increased number of CD68 positive macrophages in submucosa and muscles. Metformin or quercetin significantly reduced number of lingual macrophages with more noticeable effect for quercetin. Treatment with metformin and quercetin significantly decreased number of macrophages.

**Conclusions:**

Combined use of metformin and quercetin might help mitigate the harmful effects of radiotherapy and diabetes on lingual tissues.

## Introduction

Although radiotherapy, a type of head and neck cancer patients’ treatment, is considered a cytotoxic tumor control, it can generate protective antitumor immune responses through immunogenic cell death. Nevertheless, it has many side effects, either during or after irradiation including dry mouth, sore throat, swallowing difficulties as well as tissue fibrosis [1].

Diabetes mellitus (DM) is a widespread chronic metabolic serious health disorder and a rapidly progressing worrying disease owing to its ability to affect younger age individuals. Diabetes mellitus type 2 (T_2_DM) presents about 90% of the total prevalence of DM and is remarked by impaired insulin resistance in addition to βpancreatic cell malfunction resulting in hyperglycemia [2]. Additionally, it adversely affects carbohydrate, lipid, and protein metabolism [3]. Chronic hyperglycemia may be associated with some oral alterations such as reduced salivary flow, periodontal disease, burning mouth, taste alterations, delayed healing, infections and halitosis [4].

Alternative medicines as plant-based drugs have been recommended by the world health organization (WHO) for the treatment of DM [3]. Due to their minimum side effects and low costs, the value of alternative medicines has raised researchers’ attention in traditional herbal medicine; even when their active ingredients are obscure. Metformin, a natural product, is extracted from the plant *Galega officinalis*. It is considered as the first option for managing diabetic patients due to its superior hypoglycemic effect without affecting insulin secretion [5]. Quercetin is one of the flavonoids extracted from the fruits of pharmaceutical plants, such as *Vaccinium oxycoccos* and *Phyllanthus emblica*. It has notable anti-hyperglycemic effects with lower side effects compared to metformin [3]. Accordingly the aim of this study was to detect whether using metformin and/or quercetin will improve gamma-irradiation induced tongue toxicity in diabetic rats or not.

## Methods

### Animals & grouping

The experiment was conducted in compliance with the protocol approved by the Research Ethics Committee of the National Center for Radiation Research and Technology (REC-NCRRT), Egyptian Atomic Energy Authority, under the serial number 26 A / 22. Thirty five male albino rats weighting 200 ± 20 g. and approximately 8 weeks old were used and kept in polypropylene/stainless steel cages having dimensions of 54, 37 and 27 cm, and submitted to suitable ventilation, humidity max 55%, controlled temperature (25 max 28 °C) and light/dark regime (12 h of light/dark cycle) with pellet diet and drinkable water *ad libitum*.

Animals were randomly divided into five groups (*n* = 7): group NOR negative control (no diabetes, no radiation and no treatment), group DR positive control; rats were subjected to streptozotocin (STZ) injection followed by gamma-irradiation, group DRM; rats were subjected to STZ injection then gamma-irradiation followed by metformin application, group DRQ; rats were subjected to STZ injection followed by gamma-irradiation then quercetin application, group DRMQ; rats were subjected to STZ injection followed by gamma-irradiation then metformin and quercetin application. Rats were euthanized once 24 h after the last treatment dose by an over dose anesthesia (Ketamine).

### Diabetes mellitus induction

Rats of groups DR, DRM, DRQ and DRMQ were fasted overnight before the DM induction using STZ. Afterwards, they were intra-peritoneally injected by 35 mg/kg body weight STZ (dissolved in 0.1 M citrate buffer, pH 4.5) once at the beginning of the study. Then, they were fed 4 h after STZ injection to avoid the expected hypoglycemic shock [6]. Rats of NOR group received 0.1 ml citrate buffer& distilled water. The fasting blood glucose level (mg/dL) of different experimental groups was recorded 3 days after STZ injection using tail blood sampling with a glucometer (Perfecta, Granzia) and animals with blood glucose level above 250 mg / dl were defined as diabetic and included in the study.

### Gamma-irradiation

Rats of groups DR, DRM, DRQ and DRMQ were subjected to 2 Gy of whole body gamma-irradiation twice; one week and two weeks after STZ injection [7]. Irradiation was performed at The National Center for Radiation Research and Technology (NCRRT), using Gamma cell 40 (Cs 137) at dose rate of 0.37 Gy/min.

### Metformin and quercetin application

Rats of groups DRM, DRQ and DRMQ were treated with 200 mg/kg body weight metformin [8] (Sigma-Aldrich, USA), 30 mg/kg body weight quercetin [9] (Sigma-Aldrich, USA) or both, respectively dissolved in 0.1 ml distilled water and applied orally daily for 4 weeks starting 48 h after the second dose of gamma-irradiation.

### Blood glucose level

At the end of the experiment and before euthanasia, the fasting blood glucose level (mg/dL) of different experimental groups was recorded using tail blood sampling with a glucometer.

### Histological procedures

Specimens of tongue were fixed in 10% formalin and embedded in paraffin. Five µm thickness sections were cut and stained with hematoxylin and eosin (H&E) and CD68 immunohistochemical staining (Rabbit polyclonal anti-CD68 antibodies). Histopathological changes of the tongue were observed under light microscope. Histomorphometric analysis of the length, diameter (at base & apex) and taste buds of different lingual papillae as well as epithelial, keratin and lamina propria thickness and number of CD68 positive cells were evaluated in 10 histological fields (x400) then randomly captured with a digitized image analysis system using the software Leica Qwin 500.

### Statistical analysis

Data were subjected to one way analysis of variance (ANOVA) and expressed as mean and standard deviation and multiple range tests was used when differences among groups were significant using Statgraphics Centurion XVI software, Statpoint Technologies, Inc., 560 Broadview Ave. Warrenton, Virginia 20,182. The significant level was set to 0.05 for all tests.

## Results

### Blood glucose level

**Fig. 1:**
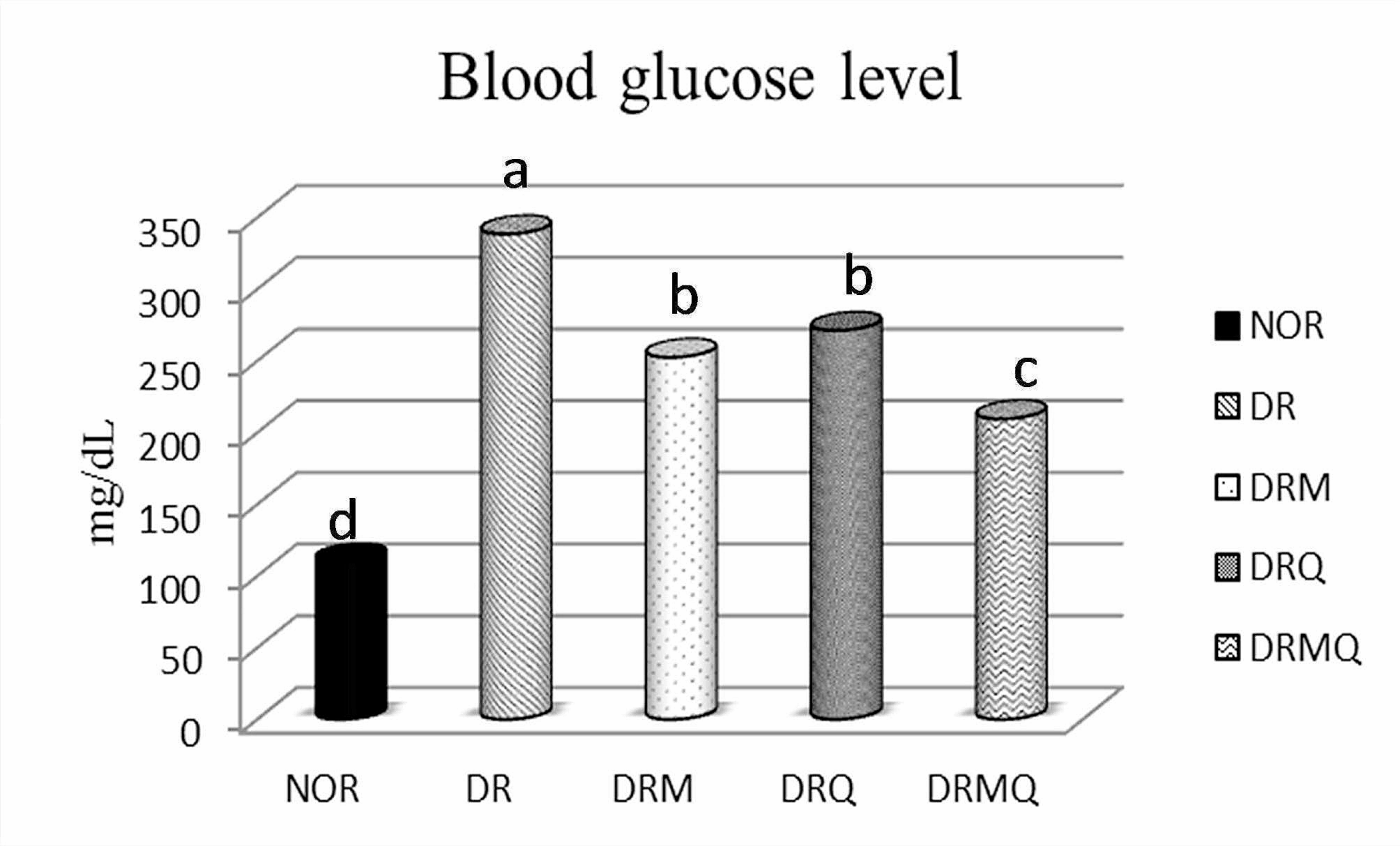
Chart represented the mean blood glucose level (mg/dL) of different experimental groups at the end of the experiment

As shown from Fig. 1, group (NOR) showed the least mean blood glucose level BGL that was highly significant compared to other groups. On contrast, group DR presented the highest mean BGL among groups with high significance compared to other groups. Regarding groups DRM and DRQ, they both showed significantly lower BGL compared to DR group however they were still significantly higher compared to the normal and combined treatment groups with insignificance in between. The BGL of group DRMQ was significantly lower compared to DR, DRM and DRQ groups, whereas higher compared to NOR group.

### Histopathological examination

**Fig. 2:**
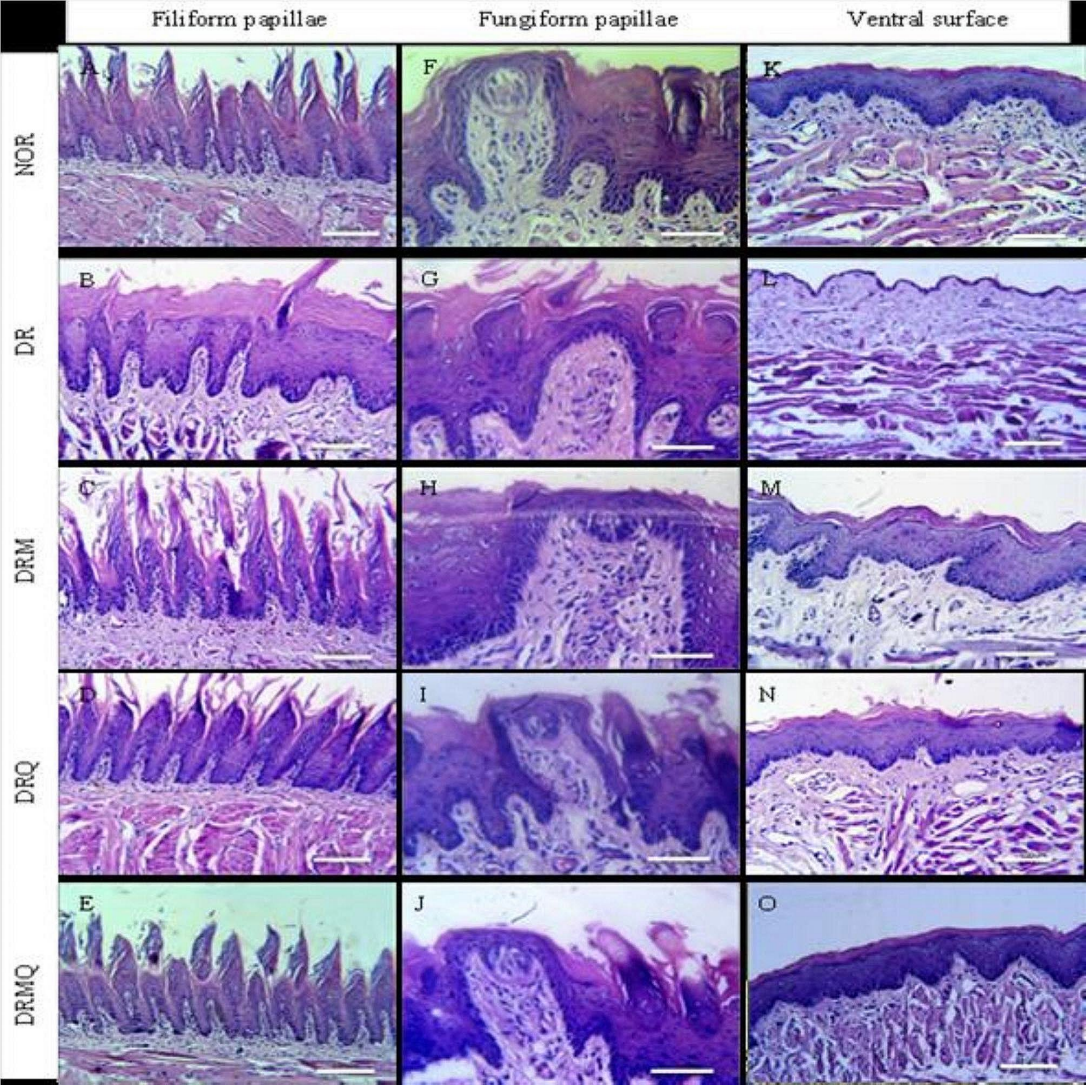
Photomicrographs of the tongue of different experimental groups (**A**, **B**, **C**, **D** and **E**) represented the filiform papillae; (**F**, **G**, **H**, **I** and **J**) represented the fungiform papillae and (**K**, **L**, **M**, **N** and **O**) represented the ventral surface

**Fig. 3:**
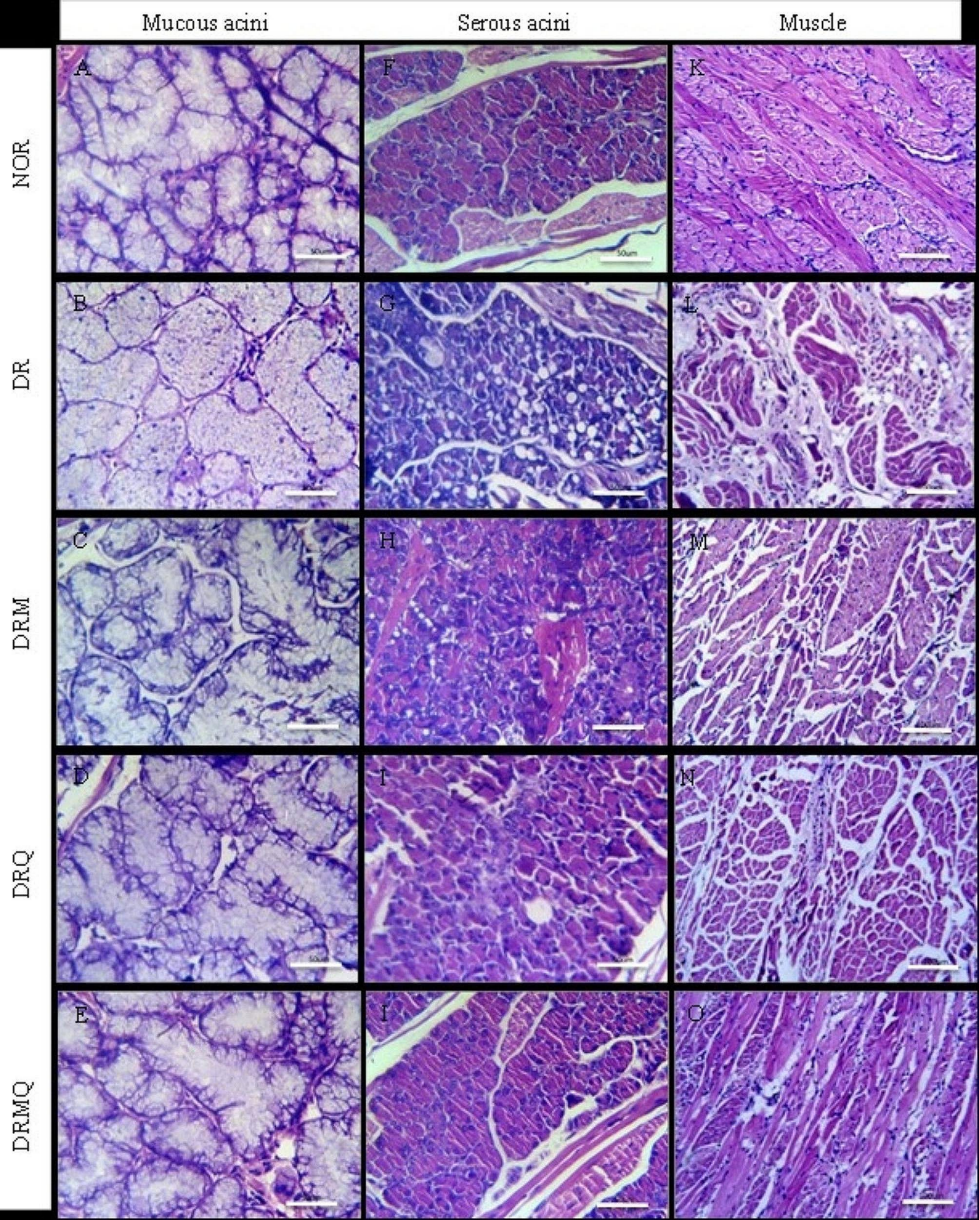
Photomicrographs of the tongue of different experimental groups (**A**, **B**, **C**, **D** and **E**) represented the mucous acini; (**F**, **G**, **H**, **I** and **J**) represented the serous acini and (**K**, **L**, **M**, **N** and **O**) represented the muscle

Examination of tongues in NOR group revealed stratified squamous epithelium with numerous and well-arranged tongue papillae covered with a regular thin keratin layer. The filiform papillae were long, thin and cone-shaped with tapering tips and well-defined secondary connective tissue. Normal fungiform papillae appeared in between filiform papillae with their characteristic dome-shape and wide well-formed connective tissue containing well-defined taste buds in the middle of their upper surfaces. The lamina propria was merged to the underlying muscle layer and consisted of loose connective tissue containing many blood vessels. The ventral surface was covered with stratified squamous epithelium that showed thin keratin layer without lingual papillae. The underlying lamina propria composed of connective tissue containing many capillaries (Fig. 2: A, F& K). Normal striated muscle fibers forming bundles running in different directions were found. Lingual salivary glands appeared between muscle bundles of the root of the tongue. Serous acini were formed of pyramidal serous cells with eosinophilic cytoplasm and basal nucleus, additionally mucous acini were formed of mucous cells with large foamy cytoplasm (Fig. 3: A, F& K).

Tongues of group DR showed generalized reduction of epithelial height, increased keratinization and shortened filiform papillae along with disappearance of secondary connective tissue. Multiple complete atrophied areas of the papillae were also seen. The broad apex of fungiform papillae was lost and became more pointed with complete taste buds degeneration. The lamina propria increased in thickness and became infiltrated with many inflammatory cells. Extreme thinning of the epithelium of the ventral surface was revealed accompanied with loss of covering keratin. The underlying connective tissue showed an increase in collagen deposition and inflammatory infiltrate (Fig. 2: B, G& L). The underlying muscle layer displayed atrophied fibers separated with wide connective tissue septa infiltrated with inflammatory cells. Many aggregates of fat cells were found between the muscle bundles. Serous acini exhibited variable sized cytoplasmic vacuolization with loss of acinar outline whereas mucous acini lost their cytoplasmic foamy appearance (Fig. 3: B, G& L).

Filiform papillae lengths of tongues of DRM group were uneven and the most were short. Some of them had irregularly aligned basal cells, rounded tips and covered with relatively thick keratin layer. Fungiform papillae had barrel shape of short lengths and wide bases. Moreover, taste buds showed slight degenerative changes. The lamina propria increased in thickness with more collagen deposition. The ventral surface showed irregular basement membrane of variable epithelial thickness covered with thick keratin layer. Wide underlying connective tissue with increased collagen deposition was observed (Fig. 2: C, H& M). The underlying muscle fibers showed slight restoration; had a preserved structures, running in different directions with separation of muscle fibers. Preserved outline and structures of the serous acini with many small cytoplasmic vacuoles was revealed. Mucous acini had a relatively normal structure with slight degenerative changes in the center of some of them (Fig. 3: C, H& M).

Tongues of group DRQ showed well-arranged and tapered filiform papillae but of reduced length. The majority of the papillae were covered by thin layer of keratin. Fungiform papillae showed their characteristic dome shape but slightly distorted and taste buds were preserved on its upper surface that had a thin layer of keratin covering. The lamina propria was normal in structure and length and merged with the underlying muscle layer smoothly. The ventral surface was formed of irregular thickness of stratified squamous epithelium covered with uniform thin layer of keratin (Fig. 2: D, I& N). The muscle layer showed longitudinal and transverse muscle fibers running in different directions. The muscle fibers were smaller in size with increased inter muscular connective tissue septa. The structure of both serous and mucous acini of lingual salivary glands was preserved (Fig. 3: D, I& N).

Tongue specimens of group DRMQ exhibited numerous thread-like filiform papillae of normal size and orientation covered with a thin keratin layer. Fungiform papillae appeared dome-shaped with normal size and preserved taste buds on its upper surface which was covered by thin keratin layer. Normal lamina propria separating the epithelium and the underlying muscle layer was detected. The ventral surface composed of keratinized stratified epithelium of almost uniform thickness covered with thin smooth keratin layer (Fig. 2: E, J& O). The muscle fibers had a normal structure similar to the control and arranged in several directions. The serous and mucous acini showed preserved structures and outline similar to control (Fig. 3: E, J& O).

Data in Table (1) represented the papillae length and diameter at base of filiform papillae all over the tongue, in addition to papillae length, diameter at base and apex and diameter of taste buds of fungiform papillae as well as thickness of epithelium, keratin and lamina propria of different groups. Regarding the length of filiform papillae at tip, anterior 2/3 and posterior 1/3 of group DR, data revealed significant reduction of length compared to the control. Groups DRM, DRQ and DRMQ showed significantly higher papillary length compared to group DR. Moreover, group DRMQ showed higher papillary length compared to each one alone.

**Table 1:** Morphometric analysis of filiform papillae, fungiform papillae, epithelium, keratin layer and lamina propria of different experimental groups

	Filiform at tip	Filiform at anterior 2/3	Filiform at posterior 1/3	
Group	PL (µm)			
NOR	330.03 ± 20.61 a	295.63 ± 7.33 a	240.78 ± 14.62 a	
DR	175.86 ± 18.42 e	143.88 ± 8.99 e	138.42 ± 11.23 d	
DRM	208.33 ± 8.41 d	202.25 ± 14.99 d	190.70 ± 14.17 c	
DRQ	240.30 ± 23.17 c	221.55 ± 15.93 c	191.98 ± 12.11 c	
DRMQ	281.35 ± 27.24 b	263.42 ± 18.04 b	225.60 ± 16.99 b	
*P* value	0.0000	0.0000	0.0000	
Group	DAB (µm)			
NOR	87.39 ± 6.58 a	94.79 ± 7.93 a	100.94 ± 5.32 a	
DR	66.38 ± 7.30 c	73.10 ± 6.05 c	85.10 ± 3.30 d	
DRM	71.58 ± 3.65 bc	87.27 ± 6.91 b	89.67 ± 6.85 cd	
DRQ	73.49 ± 7.49 b	86.56 ± 8.48 b	92.32 ± 4.17 bc	
DRMQ	85.22 ± 7.09 a	91.35 ± 5.32 ab	96.10 ± 8.27 ab	
*P* value	0.0000	0.0000	0.0000	
	Fungiform papillae			
Group	PL (µm)	DAB (µm)	DAA (µm)	DTB (µm)
NOR	201.68 ± 8.31 a	130.96 ± 8.20 a	148.31 ± 1.64 a	40.37 ± 2.68 d
DR	155.34 ± 2.82 c	113.29 ± 7.38 b	45.84 ± 1.45 d	0.00 ± 0.00 a
DRM	182.37 ± 8.04 b	133.68 ± 8.22 a	115.59 ± 3.54 c	49.96 ± 5.71 e
DRQ	185.62 ± 8.70 b	125.95 ± 5.18 a	120.90 ± 8.75 c	34.61 ± 1.88 bc
DRMQ	191.29 ± 7.86 ab	128.02 ± 4.56 a	132.22 ± 7.03 b	38.59 ± 2.10 cd
*P* value	0.0002	0.0359	0.0000	0.0000
	The dorsal tongue surface			
Group	ET (µm)	KT (µm)	LPT (µm)	
NOR	121.89 ± 9.89 a	33.57 ± 2.51 a	47.70 ± 5.29 c	
DR	76.19 ± 10.10 e	52.86 ± 4.11 c	64.37 ± 9.46 a	
DRM	96.21 ± 7.80 c	43.14 ± 4.05 b	54.94 ± 6.81 b	
DRQ	88.92 ± 8.37 d	41.73 ± 2.85 b	42.37 ± 2.78 d	
DRMQ	106.33 ± 6.37 b	33.99 ± 5.26 a	50.52 ± 3.51 c	
*P* value	0.0000	0.0000	0.0000	

PL; papillae length, DAB; diameter at base, DAA; diameter at apex, DTB; diameter of taste buds, ET; epithelial thickness, KT; keratin thickness, LPT; lamina propria thickness. Different letters mean significant difference at *p* < 0.001

About the diameter at base of filiform papillae all over the tongue, all groups exhibited significantly lower values compared to control except group DRMQ that showed insignificant difference. Groups DRM, DRQ and DRMQ represented significant higher diameter at base as compared to group DR however, the difference was insignificant with Group DRM in filiform papillae at tip and posterior 1/3. Diameter at base in group DRMQ was higher than in groups DRM and DRQ but the difference was significant with filiform papillae at tip.

The length of fungiform papillae significantly decreased in all groups as compared to control except for group DRMQ where the difference was insignificant. Groups DRM, DRQ and DRMQ showed significantly higher diameter at base as compared to group DR with no significant differences in between. The diameter at apex of fungiform papillae of control group was significantly higher than all groups; on the other hand, it was significantly higher in groups DRM, DRQ and DRMQ as compared to group DR. Additionally, group DRMQ exhibited a significantly higher diameter at base compared to either group DRM or DRQ that showed no significant difference in between. Concerning the diameter of fungiform taste buds, group DR showed completely degenerated taste buds. For group DRQ, the diameter of taste buds was significantly smaller compared to groups NOR and DR. On contrary, group DRM showed a significantly higher diameter compared to groups NOR and DR. For group DRMQ, the diameter was significantly higher than group DR but similar to control.

The measured epithelial thickness significantly decreased in all groups compared to control but it was significantly higher in groups DRM, DRQ and DRMQ compared to group DR. Among treatment groups, the highest epithelial thickness was obtained by group DRMQ followed by group DRM then group DRQ with significant difference in between. The keratin thickness of group DR was significantly higher than control. For groups DRM and DRQ, the keratin thickness was significantly lower than group DR but still significantly higher than the control. Group DRMQ exhibited significantly lower keratin thickness compared to groups DR, DRM and DRQ though it was comparable to control. The thickness of lamina propria in group DR was significantly higher than group NOR. The submucosa of group DRM showed a significant reduction in its thickness compared to group DR but still significantly higher than control. Quercetin treatment in group DRQ induced a significant reduction of submucosa thickness compared to control. The thickness of submucosa in group DRMQ showed no significant difference in comparison to control.

### CD68 immunoreactivity

**Fig. 4:**
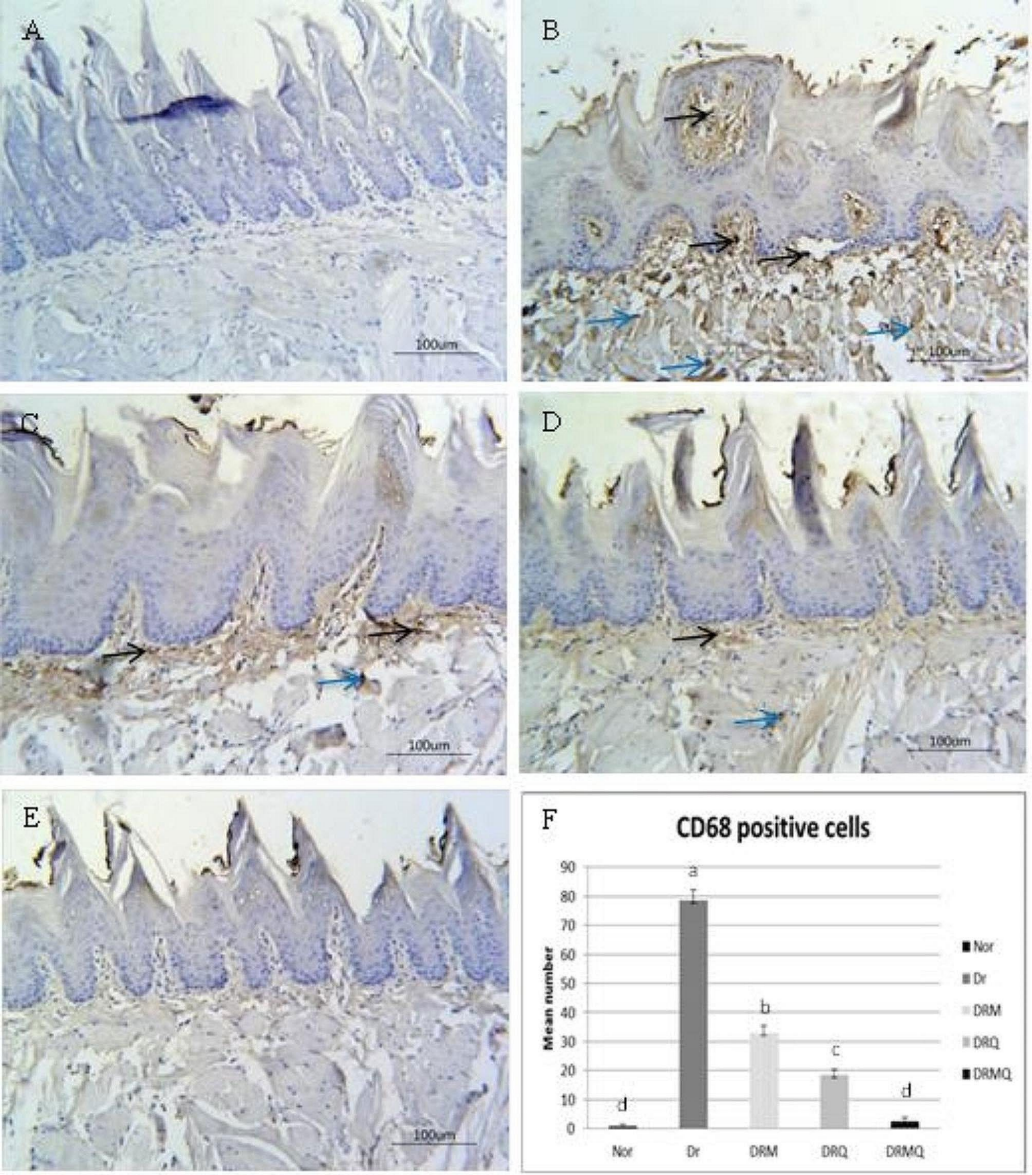
Photomicrographs of CD68 expression in the tongue of different experimental groups. **A**; NOR group with negative expression in submucosa and muscles. **B**; DR group showed severe expression in both submucosa (black arrow) and muscles (blue arrow). **C**; DRM group with moderate expression in submucosa (black arrow) and mild expression in muscles (blue arrow). **D**; DRQ group with mild expression in submucosa (black arrow) and mild expression in muscles (blue arrow). **E**; DRMQ group showed very mild expression in submucosa and negative expression in muscles. **F**; Chart represented the mean number of CD68 positive cells. Different letters mean significant difference at *p* < 0.001

The tongue sections of control group exhibited negative expression of CD68 positive macrophages in both submucosa and muscle layer (Fig. 4: A). DR group showed marked increase in the number of macrophages in submucosa and muscles fibers (Fig. 4: B). Both DRM and DRQ groups revealed an obvious decrease in lingual macrophages in submucosa and muscle layer (Fig. 4: C& D). For DRMQ group, the submucosa and muscle layer of the tongue showed almost negative expression of CD68 positive macrophages similar to control (Fig. 4: E). The mean number of CD68 positive cells significantly increased in DR group as compared to control. Metformin treatment seemed to reduce the number of CD68 positive cells compared to DR group however it was significantly higher compared to control. Quercetin induced a significant decrease in CD68 positive cells compared to DR and DRM groups but still significantly higher than control. Metformin combined with quercetin significantly reduced the number of CD68 positive cells in comparison to other groups and became comparable to control with no significance in between (Fig. 4: F).

## Discussion

The tongue, a vital organ that has multiple essential physiological functions, is the mirror of general health or disease especially its filiform papillae [10]. Diabetes mellitus is the fifth most widespread chronic condition and the sixth most common cause of death amongst elderly. The morphologic and cytomorphometric alterations in the buccal mucosa of diabetic patients can be attributed to diabetes mellitus itself [4]. Streptozotocin (STZ) is being used for DM induction due to its potential to selectively destruct the β-cell in pancreatic islets and impair insulin secretion [6]. It simulates diabetes mellitus type 1 (T_1_DM) when being used at high doses and type 2 (T_2_DM) when being used at low doses [11].

Radiotherapy of diabetic patients due to head and neck tumor is damaging to the oral tissues. Gamma radiation induces defective alterations in the tongue as well as the serous acini of the lingual minor salivary glands [12]. Development of new drugs for the management of diabetes is needed for better safety as the standard therapeutic drugs have many adverse effects, thus traditional medicine using medicinal plants is commonly used by clinicians for the treatment of diabetes [10].

In our study, the blood glucose level of irradiated diabetic rats was abnormally high. Treatment with either metformin or quercetin alone significantly reduced the glucose level besides; the combined treatment significantly reduced it although all treatments did not reach the normal level. Also, diabetes and gamma radiation induced multiple histological alteration of the tongue. The tongue showed decreased epithelial thickness and papillae height, distortion of lingual papillae with complete atrophy in many areas, loss of fungiform taste buds and increased keratinization. The muscular layer revealed some atrophied muscle fibers with wide connective tissue septa infiltrated with inflammatory cells with aggregates of fat cells. The mucous acini lost its foamy appearance while serous exhibited cytoplasmic vacuolization with loss of the outline.

Similar changes were reported by **Fakhr et al.** [13] and **Khalil and Nagui** [14] in tongues of diabetic rats. The filiform papillae were shorter, distorted with hyperkeratosis while the fungiform appeared slightly elongated with abnormal taste buds. Loss of normal connective tissue papillae was also detected. In STZ induced diabetic mice model, the pancreatic islets were infiltrated with inflammatory cells with significant increment of IL-1 and TNF- α cytokines [15]. The sensitivity of filiform papillae to damage than fungiform ones could be attributed to their high metabolic activity [10]. The above-mentioned changes could be related to chronic inflammation, changes in innervations and microvasculature secondary to diabetes [16]. Hyperkeratosis associated with diabetes was explained by increased gene expression of keratin associated proteins and keratin complexes [17]. Taste buds affection probably caused by neuropathy affecting function of taste nerves and/or microangiopathy affecting taste buds [18]. The alterations of lingual salivary glands were in accordance with **Hassan and Alqahtani** [19] who reported decreased sized acini with loss of normal arrangement and indefinite lumen of the parotid glands of alloxan induced diabetes in dogs.

Also, similar results were reported by **El-Haddad and Metwaly** [12] regarding the effect of gamma radiation on tongue. Decreased connective tissue underlying the dorsal surface of the tongue containing inflammatory cells was detected. Fragmented areas in the muscle bundles in addition to isolated fatty degeneration were also found out. The mucous acini lost their outline with hyaline cytoplasmic appearance while the serous acini appeared small, distorted with heterogenicity of staining. **El-Rouby and El-Batouti** [20] noticed disrupted architecture of basal cells, some areas of keratin loss, mild chronic inflammatory cell infiltrate and areas of degeneration in the underlying lamina propria in gamma-irradiated rat’s tongue. Salivary gland damage in response to gamma radiation could be related to direct injury of micro vascular endothelial cells with reduced micro vessel density [21].

Antioxidant enzymes in our body protect us against free radicals. However, when reactive oxygen species (ROS) overwhelm the antioxidant enzymes, it results in a state known as oxidative stress [22]. It is believed that most of the diabetic complications are related to an increase in oxidative stress in diabetic patients [23].

Metformin (dimethylbiguanide) has become one of the most favorite medications used to control T_2_DM and prescribed either in monotherapy or combined with other anti-hyperglycemic drug [24]. Moreover, metformin has an insulin sensitizing effect that affects almost all tissues including skeletal muscle, the adipose tissue, liver, endothelium and ovary [25]. In addition, metformin has been proven to have multifunctional profiles including anti-inflammatory and anti-cancer actions as well as cardiovascular protection [26].

In our study, metformin administration significantly reduced the blood glucose level as compared to irradiated diabetic group. Moreover, it provided little restoration for the lingual papillae and its taste buds with more restoration for tongue minor salivary glands and muscles however; it did not reach the normal structure. Similar result was obtained by **Cheng et al.** [27] who reported a reduction of blood glucose concentration with metformin treatment. In addition, metformin slightly preserved the skeletal muscles of diabetic rats with muscle fiber separation, some apoptotic nuclei and moderate amount of collagen fibers between muscle fibers [28]. **Chakraborty et al.** [29] assigned the histological improvement to the ability of metformin to restore the antioxidant status thus reduction of the oxidative stress. Metformin improves the negative effects of diabetes and gamma radiation. It was found that metformin treatment before irradiation improved the histological alterations and inflammation of the intestinal mucosa [30], diminished histopathological alterations of cardiac muscle [31] and suppressed chronic oxidative damage in mice bone marrow stem cells [32]. The radio-protective activity of metformin is based on several mechanisms such as; regulation of the cellular metabolism, diminish ROS production, scavenging free radicals directly or via the stimulation of antioxidant enzymes and reduction of stem cell senescence [32].

The anti-inflammatory effect of metformin against diabetes and gamma irradiation as indicated by reduced CD68 expression was in accordance with **Han et al.** [15] who proved that metformin inhibited the pro-inflammatory cytokines, such as IL-1 and TNF-α, in the pancreatic tissues of STZ induced diabetic mice that may partially explained the amelioration of the histopathological changes due to diabetes. Also, **Obafemi et al.** [33] found a significant reduction of hepatic and pancreatic IL-6 and TNF-α due to metformin treatment of diabetic rats. Accordingly, metformin can modulate radiation induced damage through inhibition of inflammation signaling pathways. It was found that sildenafil significantly improved the oral mucosal structure that was severely destructed after exposure to high dose of radiation through inhibition of inflammatory cell infiltration and reduction of the levels of nitric oxide, IL1β, IL6 and TNF-α [34].

Our results demonstrated that quercetin treatment was more able to restore the histopathological alterations of filiform and fungiform papillae as well as the ventral surface induced by diabetes and gamma irradiation. Although quercetin was able to improve the pathological effects on salivary glands, it did not provide a significant improvement with regard the muscles. The aforementioned results were in line with **Khalil and Nagui** [14] who found that quercetin treatment of diabetic rats restored the structure of both; filiform and fungiform papillae however, thick keratin layer persisted. The amelioration effect of quercetin could be attributed to many reasons; first, its hypoglycemic effect via pancreatic βcells regeneration might enhanced insulin secretion and sensitivity with decreased insulin resistance [35], second, quercetin might inhibited caspase 3 expression [14] as well as suppression of ATPase and hexokinase enzyme with stimulated mitochondrial function [36].

Quercetin exerts its anti-diabetic action by reducing intestinal glucose absorption, influencing lipid peroxidation, and enhancing the antioxidant enzymes activity in the body [37]. On the other hand, quercetin pretreatment of irradiated mice significantly reduced ulcers formation as well as their sizes allowing more healing capability and resulting in slightly disordered thin epithelium with maintained integrity. The effect of quercetin was explained by maintenance of proliferative activity and reduction of cellular senescence of the basal epithelial cells of the oral mucosa through up regulation of Ki67 and PCNA and down regulation of P21 and p16^[INK4A [38]]^. The restorative effect of quercetin on salivary gland architecture may be related to the enhancement of AQP5 expression, which in turn enhances salivary secretion [39].

Our data illustrated the beneficial effect of the combined use of metformin and quercetin than using each one alone. The lingual papillae appeared of normal size, shape and orientation covered with uniform layer of thin keratin with taste buds restoration. The structure of muscle fibers and both serous and mucous salivary glands was restored and was almost similar to those of the control. These results matches those obtained by **Eldamarawi et al.** [40] who found that the combined treatment of diabetic rats with metformin and quercetin reduced plasma glucose, insulin level and plasma level of IL-1 and TNF-α however they increased some enzymes activities in the skeletal muscle. The effect of this combination on the abovementioned parameters was better than each one alone and reached a level comparable to control.

## Conclusions

We have provided evidences that exposure to gamma radiation reduces epithelial height, increases keratinization, shortens filiform papillae, reduces width of fungiform papillae, increases the thickness of lamina propria and causes degradation of taste buds along with loss of acinar outline and the foamy appearance of the cytoplasm of mucous acini. On contrary, using metformin accompanied with quercetin regains normal size of filiform and fungiform papillae as well as normal thickness of keratin layer and lamina propria; additionally it preserves structures and outline of serous and mucous acini. Consequently, combined use of metformin and quercetin might help mitigate the harmful effects of radiotherapy and diabetes on tongue tissues.

## Data Availability

No datasets were generated or analysed during the current study.
