# Correction: The efficacy of using metformin and/or quercetin for amelioration of gamma-irradiation induced tongue toxicity in diabetic rats

**DOI:** 10.1186/s12903-024-04961-9

**Published:** 2024-11-09

**Authors:** Salwa Farid Ahmed, Mostafa A. Bakr, Amr H. Rasmy

**Affiliations:** Health Radiation Research Dept, National Center for Radiation Research and Technology, Egyptian Atomic Energy Authority, Cairo, Egypt

**Correction to: BMC Oral Health (2024) 24:110**

10.1186/s12903-024-03871-0

In this article [1], few of the Table 1 entries were missed in PDF. The complete Table 1 is given below.

**Table 1:** Morphometric analysis of filiform papillae, fungiform papillae, epithelium, keratin layer and lamina propria of different experimental groups

	Filiform at tip	Filiform at anterior 2/3		Filiform at posterior 1/3	
Group	PL (µm)				
NOR	330.03 ± 20.61 a	295.63 ± 7.33 a		240.78 ± 14.62 a	
DR	175.86 ± 18.42 e	143.88 ± 8.99 e		138.42 ± 11.23 d	
DRM	208.33 ± 8.41 d	202.25 ± 14.99 d		190.70 ± 14.17 c	
DRQ	240.30 ± 23.17 c	221.55 ± 15.93 c		191.98 ± 12.11 c	
DRMQ	281.35 ± 27.24 b	263.42 ± 18.04 b		225.60 ± 16.99 b	
*P* value	0.0000	0.0000		0.0000	
Group	DAB (µm)				
NOR	87.39 ± 6.58 a	94.79 ± 7.93 a		100.94 ± 5.32 a	
DR	66.38 ± 7.30 c	73.10 ± 6.05 c		85.10 ± 3.30 d	
DRM	71.58 ± 3.65 bc	87.27 ± 6.91 b		89.67 ± 6.85 cd	
DRQ	73.49 ± 7.49 b	86.56 ± 8.48 b		92.32 ± 4.17 bc	
DRMQ	85.22 ± 7.09 a	91.35 ± 5.32 ab		96.10 ± 8.27 ab	
*P* value	0.0000	0.0000		0.0000	
	Fungiform papillae				
Group	PL (µm)	DAB (µm)	DAA (µm)		DTB (µm)
NOR	201.68 ± 8.31 a	130.96 ± 8.20 a	148.31 ± 1.64 a		40.37 ± 2.68 d
DR	155.34 ± 2.82 c	113.29 ± 7.38 b	45.84 ± 1.45 d		0.00 ± 0.00 a
DRM	182.37 ± 8.04 b	133.68 ± 8.22 a	115.59 ± 3.54 c		49.96 ± 5.71 e
DRQ	185.62 ± 8.70 b	125.95 ± 5.18 a	120.90 ± 8.75 c		34.61 ± 1.88 bc
DRMQ	191.29 ± 7.86 ab	128.02 ± 4.56 a	132.22 ± 7.03 b		38.59 ± 2.10 cd
*P* value	0.0002	0.0359	0.0000		0.0000
	The dorsal tongue surface				
Group	ET (µm)	KT (µm)		LPT (µm)	
NOR	121.89 ± 9.89 a	33.57 ± 2.51 a		47.70 ± 5.29 c	
DR	76.19 ± 10.10 e	52.86 ± 4.11 c		64.37 ± 9.46 a	
DRM	96.21 ± 7.80 c	43.14 ± 4.05 b		54.94 ± 6.81 b	
DRQ	88.92 ± 8.37 d	41.73 ± 2.85 b		42.37 ± 2.78 d	
DRMQ	106.33 ± 6.37 b	33.99 ± 5.26 a		50.52 ± 3.51 c	
*P* value	0.0000	0.0000		0.0000	

PL; papillae length, DAB; diameter at base, DAA; diameter at apex, DTB; diameter of taste buds, ET; epithelial thickness, KT; keratin thickness, LPT; lamina propria thickness. Different letters mean significant difference at *p* < 0.001

The original article has been corrected.
